# Comparison Between Laparoscopic and Robotic Surgery in Elderly Patients With Endometrial Cancer: A Retrospective Multicentric Study

**DOI:** 10.3389/fonc.2021.724886

**Published:** 2021-09-22

**Authors:** Giacomo Corrado, Enrico Vizza, Anna Myriam Perrone, Liliana Mereu, Vito Cela, Francesco Legge, Georgios Hilaris, Tina Pasciuto, Marco D’Indinosante, Eleonora La Fera, Camilla Certelli, Valentina Bruno, Stylianos Kogeorgos, Francesco Fanfani, Pierandrea De Iaco, Giovanni Scambia, Valerio Gallotta

**Affiliations:** ^1^Dipartimento Scienze della Salute della Donna, del Bambino, e di Sanità Pubblica, Ginecologia Oncologica, Fondazione Policlinico Universitario A. Gemelli IRCCS, Rome, Italy; ^2^Department of Experimental Clinical Oncology, Gynecologic Oncology Unit, IRCCS “Regina Elena” National Cancer Institute, Rome, Italy; ^3^Division of Oncologic Gynaecology, IRCCS Azienda Ospedaliero-Universitaria di Bologna, University of Bologna, Bologna, Italy; ^4^Obstetrics and Gynecological Department, Santa Chiara Hospital, Trento, Italy; ^5^Department of Obstetrics and Gynecology, University of Pisa, Pisa, Italy; ^6^Department of Obstetrics and Gynecology, Division of Gynecology, “F. Miulli” General Hospital, Bari, Italy; ^7^2^nd^ Department of Gynecologic Oncology, Hygeia Hospital, Marousi, Athens, Greece; ^8^Department of Obstetrics and Gynecology, Division of Gynecologic Oncology, Stanford University Hospital, Stanford, CA, United States; ^9^Research Core Facilty Data Collection G-STeP, Fondazione Policlinico Universitario Agostino Gemelli IRCCS, Rome, Italy; ^10^Dipartimento Scienze della Salute della Donna, del Bambino, e di Sanità Pubblica, Ginecologia Oncologica, Fondazione Policlinico Universitario A. Gemelli—IRCCS, Università Cattolica del Sacro Cuore, Rome, Italy

**Keywords:** endometrial cancer, elderly patients, laparoscopic surgery (LS), robotic surgery, minimally invasive surgery (MIS)

## Abstract

**Introduction:**

Elderly endometrial cancer (EEC) patients represent a challenging clinical situation because of the increasing number of clinical morbidities. In this setting of patients, minimally invasive surgery (MIS) has been shown to improve surgical and clinical outcomes. The aim of this study was to evaluate the peri-operative and oncological outcomes of EEC patients who had undergone laparoscopic (LS) or robotic surgery (RS).

**Materials and Methods:**

This is a retrospective multi-institutional study in which endometrial cancer patients of 70 years or older who had undergone MIS for EC from April 2002 to October 2018 were considered. Owing to the non-randomized nature of the study design and the possible allocation biases arising from the retrospective comparison between LS and RS groups, we also performed a propensity score-matched analysis (PSMA).

**Results:**

A total of 537 patients with EC were included in the study: 346 who underwent LS and 191 who underwent RS. No significant statistical differences were found between the two groups in terms of surgical and survival outcomes. 188 were analyzed after PSMA (94 patients in the LS group were matched with 94 patients in the RS group). The median estimated blood loss was higher in the LS group (p=0.001) and the median operative time was higher in the RS group (p=0.0003). No differences emerged between LS and RS in terms of disease free survival (DFS) (p=0.890) and overall survival (OS) (p=0.683).

**Conclusions:**

Our study showed that when compared LS and RS, RS showed lower blood losses and higher operative times. However, none of the two approaches demonstrated to be superior in terms of survival outcomes. For this reason, each patient should be evaluated individually to determine the best surgical approach.

## Introduction

Endometrial cancer (EC) is the most common gynecological cancer in developed countries. A relevant percentage (15-25%) of women are older than 70 years at the diagnosis and the risk of EC increases according to the age (1, 2). Elderly patients present a higher rate of negative prognostic factors and the age itself represents a risk factor to consider in the choice of the adjuvant therapy (3). In fact, in this kind of patients more aggressive and advanced cancers are often diagnosed (4). The standard treatment is surgery in the majority of the cases. However, the main problem in the management of elderly patients is the comorbidities that increase the risk of surgical complications. For this reason, it is important on the one hand to obtain the best oncological outcome through radical surgery, and on the other hand, to reduce peri- and post-operative complications and to improve recovery times after surgery.

Several studies have investigated the feasibility of minimally invasive surgery (MIS) compared with laparotomic surgery and relevant advantages in terms of surgical outcomes have been demonstrated (5–10). However, studies in which different types of MIS in elderly patients are compared are missing.

In this study we evaluated the surgical and oncological outcomes of patients of 70 years or older who had undergone laparoscopic or robotic surgery for EC.

## Material and Methods

This is a retrospective multi-institutional study that involved patients from seven Institutes: Fondazione Policlinico Universitario A. Gemelli of Rome, Regina Elena National Cancer Institute of Rome, Santa Chiara Hospital of Trento, Azienda Ospedaliero-Universitaria di Bologna, University of Pisa, “Miulli hospital” of Acquaviva delle Fonti in Bari, Hygeia Hospital, Marousi, Athens Greece. Approval to conduct the study was obtained independently from an internal review board at each participating institution. Informed consent to laparoscopic or robotic surgery was obtained from all the patients in accordance with local and international legislation (Declaration of Helsinki) (11).

### Study Design

The data refer to a period from April 2002 to October 2018. All the EC patients of 70 years or older who had undergone MIS were considered. In the majority of the centers surgeons performed both laparoscopic and robotic surgery and the surgical approach was chosen according to clinical conditions or surgeons’ preference. The robotic platforms used were Da Vinci Si or Xi (Intuitive Surgical Sunnyvale, CA). The cut-off of 70 years was based on previous studies, in which the incidence of comorbidities relevant for surgery had been considered (5, 12). All the patients were evaluated before surgery by means of a medical history, physical examination, vaginal-pelvic examination, chest X-ray, ultrasound scans, pelvic magnetic resonance imaging (MRI) or computed tomography (CT) scans. The number of relevant comorbidities was collected for each patients. Because of the retrospective nature of the study, no comorbidity scoring systems were available. Details relative to the surgical procedure and lymph node assessment [i.e. systematic lymphadenectomy or lymph node sampling or sentinel lymph node technique (SLN)] were collected in both groups. Intra-operative and post-operative complications were defined according to Common Terminology Criteria for Adverse Events (CTCAE) version 5 (13).

Adjuvant therapy was tailored to the pathologic findings at the primary surgery after multidisciplinary tumor board (gynecologic oncology, pathology, radiation oncology, medical oncology) discussion. Treatment was based on the National Comprehensive Cancer Network (NCCN) guidelines (www.nccn.org > professionals > physician_gls) as well as ESGO, and ESTRO guidelines (14). Follow-up data were recorded through phone calls, if not available from medical records. Study data were stored using REDCap electronic data capture tools hosted at Fondazione Policlinico Universitario A. Gemellli, IRCCS (https://redcap-irccs.policlinicogemelli.it/) (15, 16).

### Statistical Analysis

Patient’s characteristics were described as absolute frequency and percentage for nominal variables and as median (min-max) and mean (standard deviation) for continuous variables. For the analysis, patients were divided into two groups according to the surgical procedure adopted. We distinguished women who underwent LPS (LPS group) and those who underwent RS (RS group). Moreover, in order to assess the impact of age on LPS and RS, patients were stratified according to four age classes: 70-74 years old, 75-79 years old, 80-85 years old, more than 85 years old. Comparisons between groups were made with Mann-Whitney test or Kruskal–Wallis test for continuous variables and χ2 or Fisher exact test for nominal variables, as appropriate. The normality of continuous variables was assessed with Shapiro–Francia test. In order to assess the rule of age, BMI, comorbidity, previous abdominal surgery, FIGO stage (17), histotype, grading, presence of metastasis, surgical approach (LPS *vs* RS), operative time (OT) and adjuvant therapy on surgical complications, univariable logistic regression analyses were run to identify possible factors significantly associated with intra-operative, early post-operative (≤30 days) and late post-operative (> 30 days) complications. The parameters were selected according to their clinical relevance and results were presented as Odds ratios (95% Confidence Intervals).

Owing to the non-randomized nature of the study design and the possible allocation biases arising from the retrospective comparison between LPS and RS groups, we also performed a propensity score-matched analysis (PSMA) (18). The PSMA was used to minimize potential selection bias and compare the treatment effects by taking into account all covariates that may influence the selection of the surgical approach (19, 20) namely laparoscopy or robotic surgery. Propensity score was developed through multivariable logistic regression model adjusting for: age, body mass index, comorbidity (present/absent), previous abdominal surgery, lymphadenectomy, histotype and FIGO stage. A 1:1 ‘‘nearest neighbor’’ match without replacement was applied (21) meaning that each patient treated by robotic surgery was matched with one patient treated by laparoscopy who had the closest estimated propensity score.

Survival analysis was performed both for the whole study and PSMA population in terms of DFS and OS. DFS was defined as the time elapsed from first diagnosis to recurrence or last follow-up while OS was defined as the time from first diagnosis to death or last follow-up. Median follow-up was calculated according to the inverted Kaplan-Meier technique (22) OS and DFS curves were estimated by Kaplan-Meier product limit method (23) and compared by log-rank test (24). For PSMA population, Cox proportional hazards models (25) were applied to evaluate the impact on DFS and OS of age, BMI, comorbidity, previous abdominal surgery, FIGO stage, histotype, grading, presence of metastasis, surgical approach (LPS *vs* RS) and adjuvant therapy. The parameters were selected according to their clinical relevance. All estimates were presented with two-sided 95% Confidence Intervals (CIs). All statistical calculations were performed using the STATA software version 13.0 (Stata Corp, College Station, TX). Two-sided tests were used and the significance level was set at p< 0.05. No imputation was carried out for missing data.

## Results

A total of 537 patients with EC were included in the study: 346 who underwent laparoscopic surgery (LS) and 191 who underwent robotic surgery (RS). Each center contributed with patients: Fondazione Policlinico Universitario A. Gemelli with 130 patients (54 LS and 76 RS), Regina Elena National Cancer Institute with 168 patients (143 LS and 25 RS), Santa Chiara Hospital with 75 patients (36 LS and 39 RS), Azienda Ospedaliero-Universitaria of Bologna with 83 patients (71 LS and 12 RS), University of Pisa with 18 patients (18 RS), “Miulli hospital” with 40 patients (24 LS and 16 RS), Hygeia Hospital with 23 patients (18 LS and 5 RS).

### Patient Characteristics

Clinical and pathological characteristics are shown in **Table 1**. The median age was 76 (range 70-94) and 75 (range 70-88) years, respectively, in the LS and the RS group. The distribution of patients according to age and BMI was not normally distributed and is shown, respectively, in **Supplementary Figures S1** and **S2**. Analyses within both LS and RS groups didn’t show any significant statistical differences in terms of histology, grading, prior abdominal surgery and medical comorbidities. These results were also confirmed in a stratified analysis according to class age (**Supplementary Table S1**). The majority of the patients in both groups had FIGO stage I (81.5% and 82.2%, respectively, in the LS and the RS group). The 18% of the patients had a FIGO stage higher than II (**Table 1**).

**Table 1:** Clinical and pathological characteristics of 537 patients with endometrial cancer according to the type of surgery.

Characteristic	All cases	LPS	RS	p value
All cases	537	346	191	
Age, years				**0.001**
Mean (standard deviation)	76.3 (4.8)	76.8 (5.0)	75.3 (4.2)	
Median (min-max)	75 (70-94)	76 (70-94)	75 (70-88)	
BMI kg/m^2†^				0.059
Mean (standard deviation)	29.4 (6.0)	29.0 (5.6)	30.2 (6.5)	
Median (min-max)	28.9 (12.5-62)	28.4 (12.5-62)	29.1 (17.6-53)	
Comorbidities				0.066
0	60/528 (11.4)	40/343 (11.7)	20/185 (10.8)	
1	203/528 (38.4)	142/343 (41.4)	61/185 (33.0)	
2	144/528 (27.3)	94/343 (27.4)	50/185 (27.0)	
>2	121/528 (22.9)	67/343 (19.5)	54/185 (29.2)	
Previous abdominal surgery	188 (35.0)	130 (37.6)	58 (30.4)	0.094
FIGO stage				**0.003**
IA	258 (48.0)	165 (47.7)	93 (48.7)	
IB	181 (33.7)	117 (33.8)	64 (33.5)	
II	44 (8.2)	35 (10.1)	9 (4.7)	
IIIA	10 (1.9)	4 (1.2)	6 (3.1)	
IIIB	7 (1.3)	6 (1.7)	1 (0.5)	
IIIC	26 (4.8)	9 (2.6)	17 (8.9)	
IVA	3 (0.6)	3 (0.9)	0 (0)	
IVB	8 (1.5)	7 (2.0)	1 (0.5)	
Histotype				
Endometrioid	468 (87.2)	302 (87.3)	166 (86.9)	0.902
NEEC	69 (12.8)	44 (12.7)	25 (13.1)	
Grading				0.384
1	103/532 (19.4)	63/341 (18.5)	40/191 (20.9)	
2	272/532 (51.1)	182/341 (53.4)	90/191 (47.1)	
3	157/532 (29.5)	96/341 (28.2)	61/191 (31.9)	
Number of lymph nodes retrieved^‡^				0.476
Mean (standard deviation)	14.8 (9.7)	15.3 (9.9)	14.3 (9.4)	
Median (min-max)	13 (1-56)	14 (1-56)	13 (1-42)	
Lymph node metastasis				**0.002**
No	506 (94.2)	334 (96.5)	172 (90.1)	
Yes	31 (5.8)	12 (3.5)	19 (9.9)	

Results are presented as n (%) except where indicated. p value was calculated with two sided Pearson’s Chi Square test or Mann-Whitney U test for categorical and continuous not normally distributed characteristics respectively. Bold font highlights statistically significant difference. LPS, Laparoscopic Surgery; RS, Robotic Surgery; BMI, Body Mass Index; NEEC, Not endometrioid endometrial cancer. ^†^Information available for 522/537 patients. ^‡^Information available for 241 patients.

### Surgical Outcomes

Surgical, adjuvant and follow up characteristics are shown in **Table 2**. No significant statistical differences were found between the two groups in terms of type of surgery, intra-operative and post-operative complication and laparotomic conversion. In particular, the rate of intraoperative complications was 1.9%. Six intraoperative complications were documented in the LS group: 2 bowel injuries, 2 bladder injuries, 2 vaginal lacerations. Four intraoperative complications were documented in the RS group: 1 bladder injury, 1 iliac artery injury, 1 vaginal laceration, 1 bowel injury. All the intraoperative complications occurred in the two groups were classified as grade < 3 according the CTCAE. There were 4 grade 3 early postoperative complications: 1 bowel perforation in RS group and 1 bladder-vaginal fistula and 2 urinary site infections in LS group. Among late postoperative complications only 3 were classified as grade 3 according to the CTCAE: 2 laparocele or incisional hernia (1 in LS and 1 in RS group) and 1 bowel perforation in RS group. Furthermore, the total number of laparotomic conversions was 10: 5 in the LS group due to obesity reasons and an excessive visceral adipose tissue, 5 in the RS due to vessel lesion (2 cases), sigma infiltration (1case) and vessel involvement by the tumor (2 cases). One patient was converted from robotic to laparoscopic surgery to due obesity reasons. Lymphadenectomy was performed in 70.2% of RS compared to 38.9% of the LS (p<0.0001). Even if the number of the lymph nodes retrieved was the same in the two groups the rate of lymph nodes metastases was higher in the robotic group (p<0.002).

**Table 2:** Surgical, adjuvant and follow up characteristics of 537 patients with endometrial cancer according to the type of surgery.

Characteristic	All cases	LPS	RS	p value
All cases	537	346	191	
Surgical procedures				
TRH	7 (1.3)	6 (1.7)	1 (0.5)	0.484
TRH + BSO/MSO	509 (94.8)	327 (94.5)	182 (95.3)	
TRH ± BSO/MSO + Omentectomy	21 (3.9)	13 (3.8)	8 (4.2)	
Lymphadenectomy				**<0.0001**
Not performed	272 (50.7)	215 (62.1)	57 (29.8)	
Sentinel lymph node	21 (3.9)	0 (0)	21 (11.0)	
Pelvic	225 (41.9)	121 (35.0)	104 (54.5)	
Pelvic and aortic	19 (3.5)	10 (2.9)	9 (4.7)	
Estimated blood loss, mL^Ɨ^				0.244
Mean (standard deviation)	77 (79.6)	73.8 (56.0)	83.2 (112.1)	
Median (min-max)	50 (0-800)	50 (0-400)	50 (0-800)	
Operative time, min^ŧ^				**<0.0001**
Mean (standard deviation)	142.4 (71.4)	122.0 (60.2)	177.1 (75.6)	
Median (min-max)	130 (25-530)	110 (35-389)	170 (25-530)	
Hospital stay, days^ǂ^				**<0.0001**
Mean (standard deviation)	3.9 (2.7)	4.2 (2.7)	3.3 (2.8)	
Median (min-max)	3 (1-32)	4 (1-32)	3 (1-31)	
Laparotomic conversion*	11 (2.0)	5 (1.4)	6 (3.1)	0.184
Patients with intra-operative complication	10 (1.9)	6 (1.7)	4 (2.1)	0.768
Patients with post-operative complication within 30 days from surgery	31 (5.8)	22 (6.4)	9 (4.7)	0.434
Patients with post-operative complication beyond 30 days from surgery	17/533 (3.2)	11/344 (3.2)	6/189 (3.2)	0.988
Adjuvant therapy				0.707
No	287 (53.4)	187 (54)	100 (52.4)	
Yes	250 (46.6)	159 (46)	91 (47.6)	
Type of adjuvant therapy^†^				0.182
CHT	45/249 (18.1)	29/158 (18.4)	16/91 (17.6)	
EBRT	91/249 (36.5)	64/158 (40.5)	27/91 (29.7)	
BRT	47/249 (18.9)	30/158 (19)	17/91 (18.7)	
CHT+EBRT	33/249 (13.3)	16/158 (10.1)	17/91 (18.7)	
CHT+BRT	2/249 (0.8)	0/158 (0)	2/91 (2.2)	
EBRT+BRT	27/249 (10.8)	17/158 (10.8)	10/91 (11.0)	
CHT+EBRT+BRT	4/249 (1.6)	2/158 (1.3)	2/91 (2.2)	
Recurrences	77 (14.3)	52 (15.0)	25 (13.1)	0.539
Deaths	100 (18.6)	77 (22.3)	23 (12.0)	**0.004**
Median FU (95% CI), months^§^	46.0 (41.5-51.1)	58.6 (50.6-61.9)	36.0 (33.1-40.6)	nc

Results are presented as n (%) except where indicated. p value was calculated with two sided Pearson’s Chi Square test or Mann-Whitney U test for categorical and continuous not normally distributed characteristics respectively, except where indicated. Bold font highlights statistically significant difference. LPS, Laparoscopic Surgery; RS, Robotic Surgery; TRH, Total Radical Hysterectomy; BSO, Bilateral Salpingo-Oophorectomy; MSO, Monolateral Salpingo-Oophorectomy; CHT, Chemotherapy; EBRT, External brachytherapy; BRT, Brachytherapy; AWD, Alive with disease; NED, No evidence of disease; FU, follow-up; CI, Confidence interval; nc, not calculated. ^Ɨ^Information available for 442/537 patients. ^ŧ^Information available for 517/537 patients. ^ǂ^Information available for 486/537 patients. *One patient of 82 years old was converted from Robotic to laparoscopic surgery for obesity reason. ^†^In one case the type of adjuvant therapy was not available. ^§^Calculated with the inverse Kaplan-Meier technique.

The mean hospital stay was 4 days in LS group and 3 days in RS. This difference was statistically significant (p=0.0001). Days of hospitalization was statistically significant lower in robotic groups ranging from 75 to 85 years compared to laparoscopic groups (**Supplementary Table S2**). The absence of statistical significant differences between the two groups in terms of intra and post operative complications was also confirmed at univariable analysis (**Supplementary Table S1**).

### Analysis According to the Age Class

Clinical and pathological characteristics according to the age class are shown in **Supplementary Table S2**. As regards the surgical outcomes, no differences emerged in terms of EBL, OT, laparotomic conversions and intra-operative and post-operative complications when the age increased, although the median OT was higher in the RS group of each age class (**Supplementary Table S3**).

### Study Population After PSMA

One hundred eighty-eight were analyzed after PSMA (94 patients in the LS group were matched with 94 patients in the RS group). After matching, no differences emerged between the clinical and pathological characteristics of the two groups, (**Table 3**). Furthermore, there were no differences in terms of surgical procedures and adjuvant therapies (respectively, p=0.605 and p=0.461), as shown in **Table 4**. Although the median estimated blood loss (EBL) was higher in the LS group (p=0.001) and the median OT was higher in the RS group (p=0.0003), no differences were observed between the two groups in terms of intra-operative and post-operative complications rate (**Table 4**). Moreover, our results did not show differences in the laparotomic conversion rate (p=0.248).

**Table 3:** Clinical and pathological characteristics of 188 matched patients with endometrial cancer according to the type of surgery.

Characteristic	All cases	LPS	RS	p value
All cases	188	94	94	
Age, years				0.161
Mean (standard deviation)	74.4 (3.5)	74.9 (3.9)	73.9 (3.0)	
Median (min-max)	74 (70-87)	74 (70-87)	73.5 (70-85)	
BMI kg/m^2^				0.626
Mean (standard deviation)	29.5 (6)	29.0 (5.2)	30 (6.7)	
Median (min-max)	29 (17.6-52)	28.3 (18.8-48)	29 (17.6-52)	
Comorbidities				0.742
0	24/186 (12.9)	11/93 (11.8)	13/93 (14.0)	
1	69/186 (37.1)	38/93 (40.9)	31/93 (33.3)	
2	47/186 (25.3)	23/93 (24.7)	24/93 (25.8)	
>2	46/186 (24.7)	21/93 (22.6)	25/93 (26.9)	
Previous abdominal surgery	49 (26.1)	26 (27.7)	23 (24.5)	0.618
FIGO stage				0.106
IA	83 (44.1)	44 (46.8)	39 (41.5)	
IB	69 (36.7)	35 (37.2)	34 (36.2)	
II	10 (5.3)	8 (8.5)	2 (2.1)	
IIIA	5 (2.7)	1 (1.1)	4 (4.3)	
IIIB	2 (1.1)	1 (1.1)	1 (1.1)	
IIIC	17 (9.0)	4 (4.3)	13 (13.8)	
IVA	0 (0)	0 (0)	0 (0)	
IVB	2 (1.1)	1 (1.1)	1 (1.1)	
Histotype				0.835
Endometrioid	161 (85.6)	80 (85.1)	81 (86.2)	
NEEC	27 (14.4)	14 (14.9)	13 (13.8)	
Grading				0.679
1	32/188 (17)	14/94 (14.9)	18/94 (19.1)	
2	97/188 (51.6)	51/94 (54.3)	46/94 (48.9)	
3	59/188 (31.4)	29/94 (30.9)	30/94 (31.9)	
Number of lymph nodes retrieved^‡^				0.729
Mean (standard deviation)	15.4 (9.1)	15.7 (9.3)	15.1 (8.9)	
Median (min-max)	15 (1-42)	15 (2-39)	14 (1-42)	
Lymph node metastasis				0.058
No	168 (89.4)	88 (93.6)	80 (85.1)	
Yes	20 (10.6)	6 (6.4)	14 (14.9)	

Results are presented as n (%) except where indicated. p value was calculated with two sided Pearson’s Chi Square test or Mann-Whitney U test for categorical and continuous not normally distributed characteristics respectively. Bold font highlights statistically significant difference. LPS, Laparoscopic Surgery; RS, Robotic Surgery; BMI, Body Mass Index; NEEC, Not endometrioid endometrial cancer. ^‡^Information available for 168 patients.

**Table 4:** Surgical, adjuvant and follow up characteristics of 188 matched patients with endometrial cancer according to the type of surgery.

Characteristic	All cases	LPS	RS	p value
All cases	188	94	94	
Surgical procedures				0.605
TRH	1 (0.5)	0 (0)	1 (1.1)	
TRH + BSO/MSO	175 (93.1)	88 (93.6)	87 (92.6)	
TRH ± BSO/MSO + Omentectomy	12 (6.4)	6 (6.4)	6 (6.4)	
Lymphadenectomy				0.484
Not performed	18 (9.6)	8 (8.5)	10 (10.6)	
Sentinel lymph node	2 (1.1)	0 (0)	2 (2.1)	
Pelvic	153 (81.4)	79 (84.0)	74 (78.7)	
Pelvic and aortic	15 (8)	7 (7.4)	8 (8.5)	
Estimated blood loss, mL^Ɨ^				**0.001**
Mean (standard deviation)	82.1 (96.4)	87.8 (63.5)	75.5 (124.2)	
Median (min-max)	50 (0-800)	99 (9-400)	50 (0-800)	
Operative time, min^ŧ^				**0.0003**
Mean (standard deviation)	178.6 (75.7)	158.6 (64.1)	197.5 (81.1)	
Median (min-max)	178 (25-530)	150 (60-389)	187.5 (25-530)	
Hospital stay, days^ǂ^				**0.0002**
Mean (standard deviation)	4.1 (3.4)	4.9 (4.3)	3.3 (2.0)	
Median (min-max)	3 (1-32)	4 (1-32)	3 (1-12)	
Laparotomic conversion	7 (3.7)	2 (2.1)	5 (5.3)	0.248
Patients with intra-operative complication	6 (3.2)	3 (3.2)	3 (3.2)	1
Patients with post-operative complication within 30 days from surgery	15 (8.0)	10 (10.6)	5 (5.3)	0.178
Patients with post-operative complication beyond 30 days from surgery	10/186 (5.4)	6/94 (6.4)	4/92 (4.3)	0.538
Adjuvant therapy				0.461
No	81 (43.1)	43 (45.7)	38 (40.4)	
Yes	107 (56.9)	51 (54.3)	56 (59.6)	
Type of adjuvant therapy				0.493
CHT	22/107 (20.6)	11/51 (21.6)	11/56 (19.6)	
EBRT	27/107 (25.2)	13/51 (25.5)	14/56 (25.0)	
BRT	30/107 (28.0)	18/51 (35.3)	12/56 (21.4)	
CHT+EBRT	14/107 (13.1)	4/51 (7.8)	10/56 (17.9)	
CHT+BRT	1/107 (0.9)	0/51 (0)	1/56 (1.8)	
EBRT+BRT	11/107 (10.3)	4/51 (7.8)	7/56 (12.5)	
CHT+EBRT+BRT	2/107 (1.9)	1/51 (2.0)	1/56 (1.8)	
Recurrences	31 (16.5)	16 (17.0)	15 (16.0)	0.844
Deaths	35 (18.6)	22 (23.4)	13 (13.8)	0.092
Median FU (95% CI), months^§^	46.0 (40.7-53.4)	57.9 (48.5-70.8)	40.4 (34.8-45.8)	nc

Results are presented as n (%) except where indicated. p value was calculated with two sided Pearson’s Chi Square test or Mann-Whitney U test for categorical and continuous not normally distributed characteristics respectively, except where indicated. Bold font highlights statistically significant difference. LPS, Laparoscopic Surgery; RS, Robotic Surgery; TRH, Total Radical Hysterectomy; BSO, Bilateral Salpingo-Oophorectomy; MSO, Monolateral Salpingo-Oophorectomy; CHT, Chemotherapy; EBRT, External brachytherapy; BRT, Brachytherapy; AWD, Alive with disease; NED, No evidence of disease; FU, follow-up; CI, Confidence interval; nc, not calculated. ^Ɨ^Information available for 155/188 patients. ^ŧ^Information available for 183/188 patients. ^ǂ^Information available for 174/188 patients. ^§^Calculated with the inverse Kaplan-Meier technique.

### Survival Outcomes

No significant differences were found between the two groups regarding the rate of patients who underwent adjuvant therapy (p=0.707 and p=0.461 for the whole study and PSMA population respectively). Similarly, no significant differences were detected in terms of modality of adjuvant therapy (p=0.171 and p=0.493 for the whole study and PSMA population respectively). Median follow up was 46.0 months (95% CI: 41.5-51.1) and 46.0 months (95% CI: 40.7-53.4) for the whole study and PSMA population respectively. In this period, in the whole study population, we observed 77 recurrences: 15.0% and 13.1% had recurrence in LS and RS groups respectively (p=0.539); while in the PSMA population, we observed 31 recurrences: 17.0% and 16.6% had recurrence in LH and RH groups respectively (p=0.844) (**Tables 2** and **4**).

**Figure 1:**
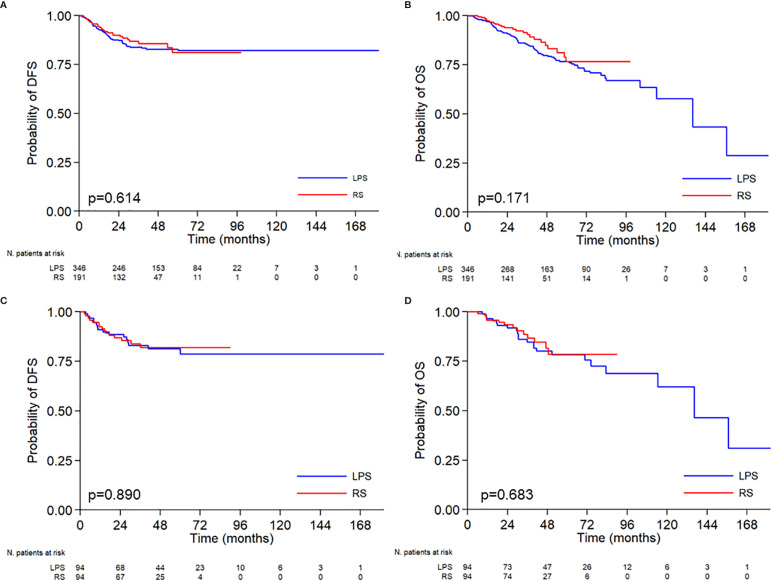
Kaplan-Meier curves relative to disease free survival-DFS **(A–C)**, and overall survival-OS **(B–D)** according to the surgical approach. Median DFS: not reached *vs* not reached. Probability of DFS at 5 years 78.8% *vs* 81.9%. Median OS: 136.2 months *vs* not reached. Probability of OS at 5 years 78.2% *vs* 78.6%.

No differences emerged between LS and RS in terms of disease free survival (DFS) (p=0.614 and p=0.890 for the whole study and PSMA population respectively) and overall survival (OS) (p=0.171 and p=0.683 for the whole study and PSMA population respectively), as shown in **Figure 1**. At the univariable analysis, there were no differences in the DFS and the OS according to the age of the PSMA patients (**Table 5**). The only variables that affected survival were, respectively, the FIGO stage for DFS and the histotype for OS.

**Table 5:** Univariable analysis of clinical, pathological and treatment characteristics of 188 matched patients with endometrial cancer according to DFS and OS.

Characteristic	Patient at risk	Disease free survival			Overall survival		
		N° events	HR (95% CI)	p value	N° events	HR (95% CI)	p value
Age	188	31	0.93 (0.83-1.04)	0.204	35	0.96 (0.87-1.05)	0.391
Age class							
70-74 years	105	20	1.00		20	1.00	
75-79 years	66	8	0.64 (0.28-1.46)	0.289	14	0.99 (0.49-2.02)	0.986
80-84 years	14	1	0.31 (0.04-2.29)	0.249	0	1.00 (empty class)	–
85+ years	3	2	3.3 (0.77-14.15)	0.107	1	1.12 (0.15-8.55)	0.909
BMI	188	31	1.02 (0.96-1.08)	0.498	35	1 (0.95-1.06)	0.942
Comorbidities							
0	24		1.00		5	1.00	
1	69		0.59 (0.21-1.61)	0.302	14	0.91 (0.32-2.55)	0.850
2	47		0.63 (0.21-1.88)	0.406	6	0.72 (0.22-2.38)	0.593
>2	46		0.70 (0.24-2.01)	0.505	10	1.13 (0.38-3.33)	0.823
Previous abdominal surgery							
No	139	23	1.00		28	1.00	
Yes	49	8	0.94 (0.42-2.09)	0.871	7	0.7 (0.3-1.62)	0.408
FIGO stage							
IA	83	7	1.00		10	1.00	
IB	69	11	2.02 (0.78-5.21)	0.147	14	1.81 (0.79-4.11)	0.158
II	10	4	5.28 (1.54-18.06)	**0.008**	3	2.21 (0.61-8.07)	0.229
IIIA	5	3	13.37 (3.45-51.85)	**<0.0001**	1	2.3 (0.29-18.11)	0.431
IIIB	2	2	29.06 (5.68-148.82)	**<0.0001**	1	7.67 (0.97-60.65)	0.053
IIIC	17	3	2.22 (0.57-8.59)	0.248	5	2.52 (0.86-7.4)	0.092
IVB	2	1	11.5 (1.38-95.54)	**0.024**	1	13.53 (1.63-112.51)	**0.016**
Histotype							
Endometrioid	161	25	1.00		22	1.00	
NEEC	27	6	1.54 (0.63-3.75)	0.343	13	3.56 (1.78-7.11)	**<0.0001**
Grading							
1	32	6	1.00		3	1.00	
2	97	10	0.48 (0.17-1.32)	0.156	12	1.12 (0.32-3.99)	0.859
3	59	15	1.34 (0.52-3.45)	0.547	20	3.08 (0.91-10.48)	0.071
Lymph node metastasis							
No	168	27	1.00		31	1.00	
Yes	20	4	1.28 (0.45-3.67)	0.643	4	1.18 (0.42-3.37)	0.754
Surgical approach							
LPS	94	16	1.00		22	1.00	
RS	94	15	0.95 (0.47-1.93)	0.890	13	0.86 (0.42-1.77)	0.683
Adjuvant therapy							
No	81	9	1.00		11	1.00	
Yes	107	22	2 (0.92-4.35)	0.080	24	1.79 (0.87-3.65)	0.111

Bold font highlights statistically significant difference. HR, Hazard Ratio; CI, Confidence Interval; BMI, Body Mass Index; NEEC, Not endometrioid endometrial cancer; LPS, Laparoscopic Surgery; RS, Robotic Surgery.

## Discussion

This study confirms the benefit of the MIS approach in elderly endometrial cancer patients (4–10). After 4 years of follow-up, the present data suggest that MIS in EEC patients is safe from an oncological standpoint in terms of comparable DFS and OS rates.

Based on our multicentric experience, we can assert that robotic and laparoscopic approach for elderly endometrial cancer patients can be well tolerated with no increase in complications. Although, the RS required longer operative time, on the other hand it showed advantages in terms of reduced blood loss and hospital stay compared to LS.

Overall, our data confirm the available lines of evidence supporting the safety of MIS. The incidence of overall post-operative complications in our cohort was 5.8%, a frequency in agreement with some previous results (4–10), without significant difference between the two groups, despite about 40% of obese patients in each group.

Since elderly patients usually present a higher comorbidity rate and a higher surgical risk, in recent years the efforts have focused on the choice of the best surgical approach for this kind of patients. The main issues related to MIS were anesthesiological: the maintenance of Trendelemburg position and the pneumoperitoneum increase abdominal pressure reducing cardiac output and respiratory movements (5, 6, 26). For this reason, the management of these patients require a close collaboration within a multidisciplinary team consisting of anesthesia, geriatric and gynecologic specialists in order to obtain a greater synergy for determining surgical indications and tailored approaches in these fragile patients (27, 28). However, the increasing expertise of the surgeons with lower operative times may reduce the relevance of these issues. Furthermore, MIS has shown good results in terms of lower complication rates and faster recovery times. Our series confirmed that MIS is associated with good post-operative results, with clinical benefits in terms of post-operative complications (29). When compared with laparotomic surgery in EC patients aged 70 years or older, RS showed a reduction of EBL, OT, complications and days of hospitalization (5, 9). Even when elderly and not elderly patients were compared, RS maintained its advantages (7) with no differences between robotic and laparotomic approaches in terms of survival (5). In the same way, LS showed better surgical outcomes when compared with a laparotomic approach. Laparotomy, in fact, was associated with a higher risk of thromboembolism, due to a longer recovery time, and higher surgical site infections rate (6). Furthermore, prolonged hospitalization times may delay the start of adjuvant therapies, compromising their efficacy. Although some recent studies compared the three different approaches (LS, RS and laparotomic surgery) according to the age of the patients, confirming an advantage of minimally invasive surgery (30, 31), studies in which the best minimally invasive approach was evaluated are missing. In our study we compared LS and RS in elderly patients (70 years and older) with EC and no differences emerged between the two surgical approaches in terms of complication rates, both intra-operative and post-operative. The LS group showed a higher median EBL, probably because of a better surgical field control with robotic arms, whereas the OT were longer in the RS group, due to docking times. In a recent study, de’ Angelis et al, who evaluated the LS and the RS in elderly patients with colorectal cancer, showed similar results with no differences in terms of surgical outcomes between the two approaches, except for a longer OT in the RS group (32). On the one hand, the increased OT in RS may be a disadvantage for elderly patients, since it may be related to a prolonged Trendelemburg position which is not reversible without the un-docking of the robot (5). On the other hand, in RS the insufflation system is different and the pressure of the pneumoperitoneum may be reduced, taking advantage from the lifting of the trocars and the abdomen during docking time.

Our results did not show a worsening of the surgical outcomes when the age increased, in agreement with Uccella et al. who demonstrated the maintenance of an advantage of LS compared with laparotomy even in patients aged 80 years or older (6) and Lowe et al. who showed a 96% successful robotic procedures in octogenarians and nonagenarians (33). Furthermore, although Walker et al. showed an increased conversion rate (from LS to Laparotomy) for each decade of age (34), our analysis did not reveal any differences in the conversion rate according to the age class. Another important aspect of surgery in elderly patients that emerged in some studies is the reduction of the lymphadenectomy rate (31), probably in order to reduce the invasiveness of the surgical procedure in this kind of patients. In our study, although the number of patients who underwent lymphadenectomy was lower when the age increased, the difference did not reach a statistical relevance. As regards survival outcomes, none of the two approaches demonstrated to be superior.

The major strengths of this study are represented by the number of patients included in the study, the PSMA and its specific focus on the role of MIS in EEC patients. Limitations include the retrospective nature of the study, which can result in underreporting adequate pre-operative frailty evaluation (28, 35) of the patients.

## Conclusions

In conclusion, thanks to the successful cooperative efforts of multiple referral Gynecologic Oncology Units, we confirmed in a large series that MIS for EEC is feasible and safe, and provides survival outcomes comparable to those obtained with open surgical approach. In particular, when compared LS and RS, RS showed lower blood losses and higher operative times. However, none of the two approaches demonstrated to be superior in terms of survival outcomes.

Several efforts should be made and prospective collaborative study are needed to provide adequate preoperative work up and availability of a dedicated multidisciplinary approach, which plays a major role in the selection of patients for the optimal management strategy in elderly endometrial cancer patients.

## Data Availability Statement

The raw data supporting the conclusions of this article will be made available by the authors, without undue reservation.

## Ethics Statement

Ethical review and approval was not required for the study on human participants in accordance with the local legislation and institutional requirements. The patients/participants provided their written informed consent to participate in this study.

## Author Contributions

Conceived and designed the study: GC and VG. Collected the data: AP, LM, VC, FL, GH, MD’I, EF, CC, and VB. Data analysis: TP. Manuscript writing: CC and GC. Manuscript revision: VG. Supervision and validation: PI, EV, FF, SK, and GS. All authors contributed to the article and approved the submitted version.

## Conflict of Interest

The authors declare that the research was conducted in the absence of any commercial or financial relationships that could be construed as a potential conflict of interest.

## Publisher’s Note

All claims expressed in this article are solely those of the authors and do not necessarily represent those of their affiliated organizations, or those of the publisher, the editors and the reviewers. Any product that may be evaluated in this article, or claim that may be made by its manufacturer, is not guaranteed or endorsed by the publisher.

## Supplementary Material

Supplementary Figure 1 Histograms of the distribution of the patients according to the age.

Supplementary Figure 2 Histograms of the distribution of the patients according to body mass index (BMI).
