# Supplementary figures and images for: Comparison Between Laparoscopic and Robotic Surgery in Elderly Patients With Endometrial Cancer: A Retrospective Multicentric Study
Source: Front Oncol. 2021 Sep 22;11:724886. doi: 10.3389/fonc.2021.724886 (PMC8493293; doi:10.3389/fonc.2021.724886)

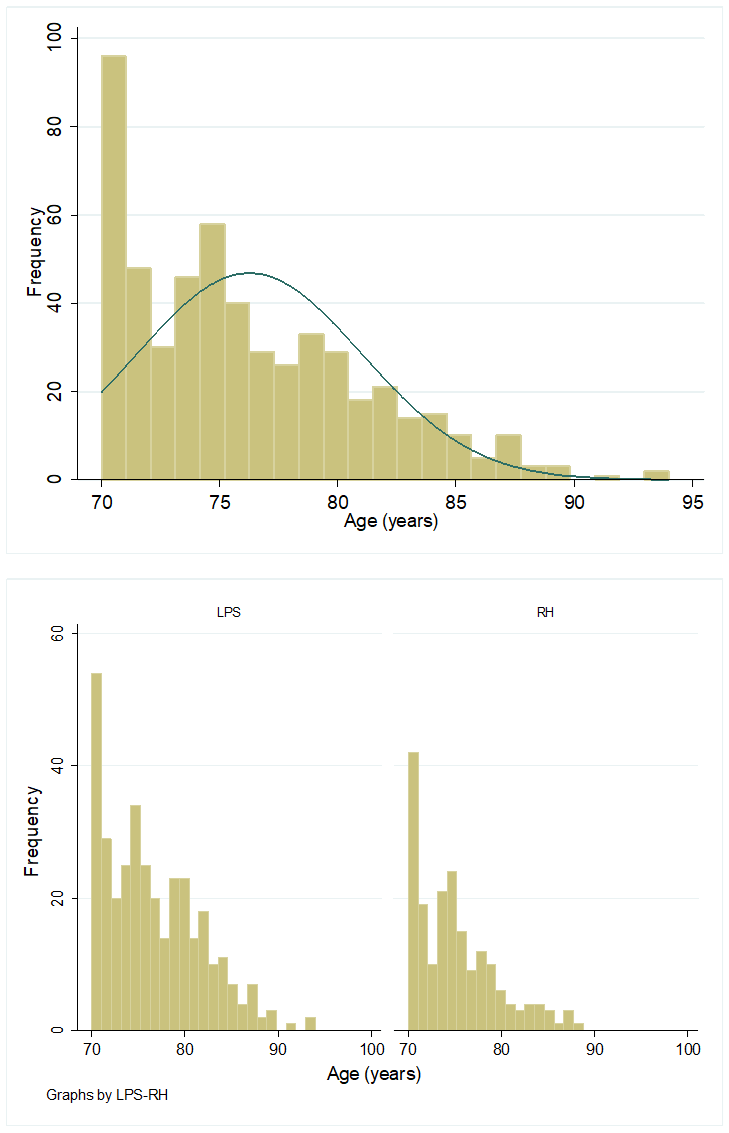

Supplement: Supplementary Figure 1 — Histograms of the distribution of the patients according to the age. [file Image_1.tif]

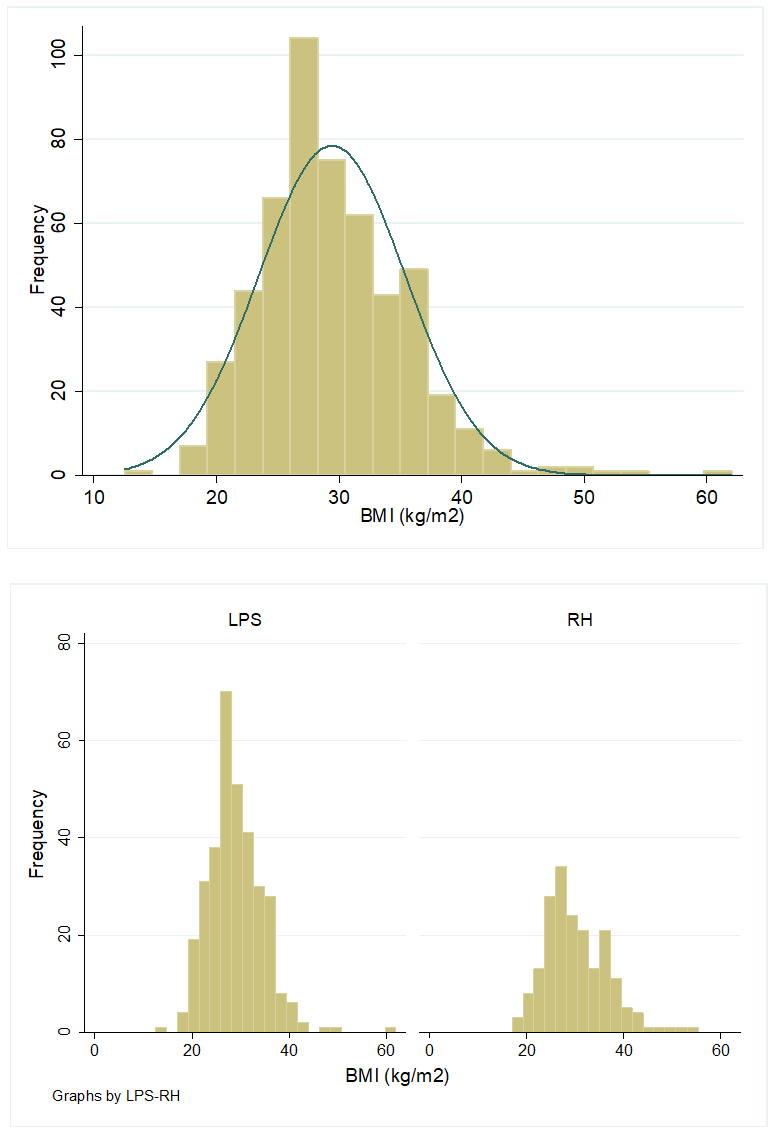

Supplement: Supplementary Figure 2 — Histograms of the distribution of the patients according to body mass index (BMI). [file Image_2.tif]
