# Supplementary material for: Comparison Between Laparoscopic and Robotic Surgery in Elderly Patients With Endometrial Cancer: A Retrospective Multicentric Study
Source: Front Oncol. 2021 Sep 22;11:724886. doi: 10.3389/fonc.2021.724886 (PMC8493293; doi:10.3389/fonc.2021.724886)
Supplement: Supplementary file 3 [file Table_1.docx]

| **Table S1.** Univariate analysis of clinical, pathological and treatment characteristics of 537 patients with endometrial cancer according to the presence of any grade of surgical complication | | | | | | | | | | | | | | |
| --- | --- | --- | --- | --- | --- | --- | --- | --- | --- | --- | --- | --- | --- | --- |
| Characteristic | Intra-operative complication | | | |  | Post-operative complication within 30 days from surgery | | | |  | Post-operative complication beyond 30 days from surgery | | | |
|  | Patients at risk | N° events | OR (95% CI) | p value |  | Patients at risk | N° events | OR (95% CI) | p value |  | Patients at risk | N° events | OR (95% CI) | p value |
| Age | 537 | 10 | 1.00 (0.89-1.14) | 0.980 |  | 537 | 31 | 1.08 (1.01-1.16) | **0.024** |  | 533 | 17 | 1.03 (0.93-1.14) | 0.552 |
| Age class |  |  |  |  |  |  |  |  |  |  |  |  |  |  |
| 70-74 years | 222 | 3 | 1.00 (Ref) |  |  | 222 | 10 | 1.00 (Ref) |  |  | 219 | 4 | 1.00 (Ref) |  |
| 75-79 years | 185 | 4 | 1.61 (0.36-7.30) | 0.535 |  | 185 | 7 | 0.83 (0.31-2.24) | 0.718 |  | 185 | 8 | 2.43 (0.72-8.20) | 0.153 |
| 80-84 years | 97 | 3 | 2.33 (0.46-11.75) | 0.306 |  | 97 | 12 | 2.99 (1.25-7.19) | **0.014** |  | 97 | 5 | 2.92 (0.77-11.12) | 0.116 |
| 85+ years | 23 | 0 | 1 (empty class) | - |  | 33 | 2 | 1.36 (0..29-6.54) | 0.695 |  | 32 | 0 | 1 (empty class) | - |
| BMI | 522 | 10 | 1.00 (0.90- 1.11) | 0.962 |  | 522 | 31 | 0.98 (0.92-1.05) | 0.571 |  | 522 | 17 | 0.98 (0.90-1.06) | 0.606 |
| Comorbidities |  |  |  |  |  |  |  |  |  |  |  |  |  |  |
| 0 | 60 | 0 | 1.00 (empty class) | - |  | 60 | 5 | 1.00 (Ref) |  |  | 60 | 2 | 1.00 (Ref) |  |
| 1 | 203 | 4 | 0.79 (0.17-3.59) | 0.761 |  | 203 | 13 | 0.75 (0.26-2.20) | 0.604 |  | 200 | 4 | 0.59 (0.121-3.31) | 0.551 |
| 2 | 144 | 3 | 0.84 (0.17-4.22) | 0.829 |  | 144 | 8 | 0.65 (0.20-2.06) | 0.462 |  | 143 | 5 | 1.05 (0.20-5.57) | 0.954 |
| >2 | 121 | 3 | 1.00 (Ref) |  |  | 121 | 5 | 0.47 (0.13-1.71) | 0.253 |  | 121 | 6 | 1.51 (0.30- 7.73) | 0.619 |
| Previous abdominal surgery |  |  |  |  |  |  |  |  |  |  |  |  |  |  |
| No | 349 | 8 | 1.00 (Ref) |  |  | 349 | 23 | 1.00 (Ref) |  |  | 346 | 8 | 1.00 (Ref) |  |
| Yes | 188 | 2 | 0.46 (0.10- 2.18) | 0.327 |  | 188 | 8 | 0.63 (0.28-1.44) | 0.272 |  | 187 | 9 | 2.13 (0.81-5.63) | 0.125 |
| FIGO stage |  |  |  |  |  |  |  |  |  |  |  |  |  |  |
| IA | 258 | 6 | 1.00 (Ref) |  |  | 258 | 18 | 1.00 (Ref) |  |  | 256 | 8 | 1.00 (Ref) |  |
| IB | 181 | 1 | 0.23 (0.03-1.95) | 0.180 |  | 181 | 9 | 0.70 (0.31-1.59) | 0.392 |  | 180 | 6 | 1.07 (0.36-3.13) | 0.903 |
| II | 44 | 0 | 1.00 (empty class) | - |  | 43 | 1 | 0.32 (0.04-2.44) | 0.270 |  | 42 | 2 | 1.55 (0.32-7.56) | 0.588 |
| IIIA | 10 | 1 | 4.67 (0.51-42.92) | 0.174 |  | 10 | 1 | 1.48 (0.18-12.35) | 0.716 |  | 10 | 0 | 1.00 (empty class) | - |
| IIIB | 7 | 0 | 1.00 (empty class) | - |  | 7 | 0 | 1.00 (empty class) | - |  | 7 | 0 | 1.00 (empty class) | - |
| IIIC | 26 | 2 | 3.5 (0.67-18.30) | 0.138 |  | 26 | 2 | 1.11 (0.24-5.08) | 0.892 |  | 26 | 1 | 1.24 (0.15-10.32) | 0.842 |
| IVA | 3 | 0 | 1.00 (empty class) | - |  | 3 | 0 | 1.00 (empty class) | - |  | 3 | 0 | 1.00 (empty class) | - |
| IVB | 8 | 0 | 1.00 (empty class) | - |  | 8 | 0 | 1.00 (empty class) | - |  | 8 | 0 | 1.00 (empty class) | - |
| Histotype |  |  |  |  |  |  |  |  |  |  |  |  |  |  |
| Endometrioid | 468 | 9 | 1.00 (Ref) |  |  | 468 | 25 | 1.00 (Ref) |  |  | 465 | 14 | 1.00 (Ref) |  |
| NEEC | 69 | 1 | 0.99 (0.35-2.82) | 0.980 |  | 69 | 6 | 1.15 (0.67-1.96) | 0.614 |  | 68 | 13 | 1.49 (0.42-5.31) | 0.542 |
| Grading |  |  |  |  |  |  |  |  |  |  |  |  |  |  |
| 1 | 103 | 3 | 1.00 (Ref) |  |  | 103 | 5 | 1.00 (Ref) |  |  | 103 | 6 | 1.00 (Ref) | continue |
| 2 | 272 | 4 | 0.50 (0.11-2.26) | 0.366 |  | 272 | 15 | 1.14 (0.40-3.23) | 0.800 |  | 269 | 6 | 0.37 (0.12-1.17) | 0.091 |
| 3 | 157 | 3 | 0.65 (0.13-3.28) | 0.601 |  | 157 | 10 | 1.33 (0.44-4.02) | 0.609 |  | 156 | 5 | 0.53 (0.16-1-80) | 0.313 |
| Lymph node metastasis |  |  |  |  |  |  |  |  |  |  |  |  |  |  |
| No | 506 | 9 | 1.00 (Ref) |  |  | 506 | 29 | 1.00 (Ref) |  |  | 502 | 15 | 1.00 (Ref) |  |
| Yes | 31 | 1 | 1.84 (0.22-15.01) | 0.569 |  | 31 | 2 | 1.13 (0.26-4.99) | 0.868 |  | 31 | 2 | 2.24 (0.49-10.26) | 0.299 |
| Surgical approach |  |  |  |  |  |  |  |  |  |  |  |  |  |  |
| LPS | 346 | 6 | 1.00 (Ref) | 0.768 |  | 346 | 22 | 1.00 (Ref) |  |  | 344 | 11 | 1.00 (Ref) |  |
| RS | 161 | 4 | 1.21 (0.34-4.35) |  |  | 161 | 9 | 0.73 (0.33-1.62) | 0.435 |  | 189 | 6 | 0.99 (0.36-2.73) | 0.988 |
| Operative time | 517 | 10 | 1.00 (1.00-1.01) | **0.016** |  | 517 | 31 | 1.00 (1.00-1.01) | **0.002** |  | 513 | 17 | 1.00 (0.99-1.01) | 0.138 |
| Adjuvant therapy |  |  |  |  |  |  |  |  |  |  |  |  |  |  |
| No | 287 | 7 | 1.00 (Ref) |  |  | 287 | 17 | 1.00 (Ref) |  |  | 287 | 10 | 1.00 (Ref) |  |
| Yes | 250 | 3 | 0.49 (0.12-1.90) | 0.299 |  | 250 | 14 | 0.94 (0.45-1.95) | 0.873 |  | 246 | 7 | 0.81 (0.30-2.16) | 0.676 |
| Bold font highlights statistically significant difference. OR: Odds Ratio. CI: Confidence Interval. BMI: Body Mass Index. NEEC: Not endometrioid endometrial cancer. LPS: Laparoscopic Surgery. RS: Robotic Surgery. | | | | | | | | | | | | | | |

| **Table S2.** Clinical and pathological characteristics of 537 patients with endometrial cancer according to the type of surgery and the age class | | | | | | | | | | | | | | | | | | |
| --- | --- | --- | --- | --- | --- | --- | --- | --- | --- | --- | --- | --- | --- | --- | --- | --- | --- | --- |
| Characteristic | Age 70-74 years | | |  | Age 75-79 years | | |  | Age 80-84 years | | |  | Age 85+ years | | |  | p value within LPS group according to age class | p value within RS group according to age class |
|  | LPS | RS | p value |  | LPS | RS | p value |  | LPS | RS | p value |  | LPS | RS | p value |  |  |  |
| All cases | 130 | 92 |  |  | 115 | 70 |  |  | 76 | 21 |  |  | 25 | 8 |  |  |  |  |
| Age, years |  |  | 1 |  |  |  | 0.357 |  |  |  | 0.754 |  |  |  | 0.291 |  | **0.0001 *** | **0.0001 *** |
| Mean (standard deviation) | 71.9 (1.5) | 71.9 (1.4) |  |  | 71.9 (1.5) | 71.9 (1.5) |  |  | 71.9 (1.5) | 71.9 (1.5) |  |  | 71.9 (1.5) | 71.9 (1.5) |  |  |  |  |
| Median (min-max) | 72 (70-74.8) | 72 (70-74) |  |  | 72 (70-74.8) | 72 (70-74.8) |  |  | 72 (70-74.8) | 72 (70-74.8) |  |  | 72 (70-74.8) | 72 (70-74.8) |  |  |  |  |
| BMI kg/m^2^ † |  |  | 0.051 |  |  |  | 0.814 |  |  |  | 0.581 |  |  |  | 0.114 |  | 0.198 * | 0.373 * |
| Mean (standard deviation) | 29.4 (6.5) | 31 (6.7) |  |  | 29.4 (6.5) | 29.4 (6.5) |  |  | 29.4 (6.5) | 29.4 (6.5) |  |  | 29.4 (6.5) | 29.4 (6.5) |  |  |  |  |
| Median (min-max) | 28.1 (18.8-62) | 30 (17.6-53) |  |  | 28.1 (18.8-62) | 28.1 (18.8-62) |  |  | 28.1 (18.8-62) | 28.1 (18.8-62) |  |  | 28.1 (18.8-62) | 28.1 (18.8-62) |  |  |  |  |
| Comorbidities |  |  | 0.643 |  |  |  | 0.117 |  |  |  | 0.375 |  |  |  | 0.164 |  | **0.023** | 0.386 |
| 0 | 16/129 (12.4) | 11/90 (12.2) |  |  | 12/113 (10.6) | 5/67 (7.5) |  |  | 5/76 (6.6) | 4/21 (19.0) |  |  | 7/25 (28.0) | 0/7 (0) |  |  |  |  |
| 1 | 46/129 (35.7) | 26/90 (28.9) |  |  | 55/113 (48.7) | 24/67 (35.8) |  |  | 36/76 (47.4) | 9/21 (42.9) |  |  | 5/25 (20.0) | 2/7 (28.6) |  |  |  |  |
| 2 | 42/129 (32.6) | 30/90 (33.3) |  |  | 23/113 (20.4) | 14/67 (20.9) |  |  | 19/76 (25.0) | 4/21 (19.0) |  |  | 10/25 (40.0) | 2/7 (28.6) |  |  |  |  |
| >2 | 25/129 (19.4) | 23/90 (25.6) |  |  | 23/113 (20.4) | 24/67 (35.8) |  |  | 16/76 (21.1) | 4/21 (19.0) |  |  | 3/25 (12.0) | 3/7 (42.9) |  |  |  |  |
| Previous abdominal surgery | 50 (38.5) | 33 (35.9) | 0.694 |  | 48 (41.7) | 16 (22.9) | **0.009** |  | 28 (36.8) | 8 (38.1) | 0.916 |  | 4 (16.0) | 1 (12.5) | 0.810 |  | 0.118 | 0.191 |
| FIGO stage |  |  | 0.089 |  |  |  | 0.190 |  |  |  | 0.513 |  |  |  | 0.569 |  | 0.476 | 0.955 |
| IA | 70 (53.8) | 44 (47.8) |  |  | 53 (46.1) | 36 (51.4) |  |  | 32 (42.1) | 10 (47.6) |  |  | 10 (40.0) | 3 (37.5) |  |  |  |  |
| IB | 35 (26.9) | 31 (33.7) |  |  | 45 (39.1) | 22 (31.4) |  |  | 28 (36.8) | 6 (28.6) |  |  | 9 (36.0) | 5 (62.5) |  |  |  |  |
| II | 14 (10.8) | 4 (4.3) |  |  | 9 (7.8) | 3 (4.3) |  |  | 9 (11.8) | 2 (9.5) |  |  | 3 (12.0) | 0 (0) |  |  |  |  |
| IIIA | 1 (0.8) | 2 (2.2) |  |  | 3 (2.6) | 3 (4.3) |  |  | 0 (0) | 1 (4.8) |  |  | 0 (0) | 0 (0) |  |  |  |  |
| IIIB | 1 (0.8) | 1 (1.1) |  |  | 2 (1.7) | 0 (0) |  |  | 1 (1.3) | 0 (0) |  |  | 2 (8.0) | 0 (0) |  |  |  |  |
| IIIC | 4 (3.1) | 10 (10.9) |  |  | 1 (0.9) | 5 (7.1) |  |  | 3 (3.9) | 2 (9.5) |  |  | 1 (4.0) | 0 (0) |  |  |  |  |
| IVA | 2 (1.5) | 0 (0) |  |  | 0 (0) | 0 (0) |  |  | 1 (1.3) | 0 (0) |  |  | 0 (0) | 0 (0) |  |  |  |  |
| IVB | 3 (2.3) | 0 (0) |  |  | 2 (1.7) | 1 (1.4) |  |  | 2 (2.6) | 0 (0) |  |  | 0 (0) | 0 (0) |  |  |  |  |
| Histotype |  |  | 0.901 |  |  |  | 0.445 |  |  |  | 0.372 |  |  |  | 0.970 |  | 0.537 | 0.806 |
| Endometrioid | 111 (85.4) | 78 (84.8) |  |  | 99 (86.1) | 63 (90.0) |  |  | 70 (92.1) | 18 (85.7) |  |  | 22 (88.0) | 7 (87.5) |  |  |  |  |
| NEEC | 19 (14.6) | 14 (15.2) |  |  | 16 (13.9) | 7 (10.0) |  |  | 6 (7.9) | 3 (14.3) |  |  | 3 (12.0) | 1 (12.5) |  |  |  |  |
| Grading |  |  | 0.716 |  |  |  | **0.014** |  |  |  | 0.321 |  |  |  | 0.514 |  | 0.415 | 0.275 |
| 1 | 26/129 (20.2) | 15/92 (16.3) |  |  | 13/111 (11.7) | 21/70 (30) |  |  | 20/76 (26.3) | 3/21 (14.3) |  |  | 4/25 (16) | 1/8 (12.5) |  |  |  |  |
| 2 | 67/129 (51.9) | 48/92 (52.2) |  |  | 66/111 (59.5) | 30/70 (42.9) |  |  | 35/76 (46.1) | 9/21 (42.9) |  |  | 14/25 (56) | 3/8 (37.5) |  |  |  |  |
| 3 | 36/129 (27.9) | 29/92 (31.5) |  |  | 32/111 (28.8) | 19/70 (27.1) |  |  | 21/76 (27.6) | 9/21 (42.9) |  |  | 7/25 (28) | 4/8 (50.0) |  |  |  |  |
| Number of lymph nodes retrieved ‡ |  |  | 0.815 |  |  |  | 0.271 |  |  |  | **0.007** |  |  |  | 0.096 |  | **0.027 *** | 0.086 * |
| Mean (standard deviation) | 16.2 (9.8) | 15.7 (9.5) |  |  | 16.2 (9.8) | 15.7 (9.5) |  |  | 16.2 (9.8) | 15.7 (9.5) |  |  | 16.2 (9.8) | 15.7 (9.5) |  |  |  |  |
| Median (min-max) | 15 (2-39) | 13 (1-42) |  |  | 15 (2-39) | 13 (1-42) |  |  | 15 (2-39) | 13 (1-42) |  |  | 15 (2-39) | 13 (1-42) |  |  |  |  |
| Lymph node metastasis |  |  | **0.043** |  |  |  | 0.141 |  |  |  | **0.033** |  |  |  | 0.566 |  | 0.815 | 0.502 |
| No | 124 (95.4) | 81 (88.0) |  |  | 112 (97.4) | 65 (92.9) |  |  | 74 (97.4) | 18 (85.7) |  |  | 24 (96.0) | 8 (100) |  |  |  |  |
| Yes | 6 (4.6) | 11 (12.0) |  |  | 3 (2.6) | 5 (7.1) |  |  | 2 (2.6) | 3 (14.3) |  |  | 1 (4.0) | 0 (0) |  |  |  |  |
| Results are presented as n (%) except where indicated. p value was calculated with two sided Pearson's Chi Square test or Mann-Whitney U test for categorical and continuous not normally distributed characteristics respectively, except where indicated. Bold font highlights statistically significant difference. LPS: Laparoscopic Surgery. RS: Robotic Surgery. BMI: Body Mass Index. NEEC: Not endometrioid endometrial cancer. * Calculated with two sided Kruskall-Walli test (not normally distributed characteristic). † Information available for 522/537 patients. ‡ Information available for 241 patients. | | | | | | | | | | | | | | | | | | |

| **Table S3.** Surgical, adjuvant and follow up characteristics of 537 patients with endometrial cancer according to the type of surgery and the age class | | | | | | | | | | | | | | | | | | |
| --- | --- | --- | --- | --- | --- | --- | --- | --- | --- | --- | --- | --- | --- | --- | --- | --- | --- | --- |
| Characteristic | Age 70-74 years | | |  | Age 75-79 years | | |  | Age 80-84 years | | |  | Age 85+ years | | |  | p value within LPS group according to the aging classes | p value within RS group according to the aging classes |
|  | LPS | RS | p value |  | LPS | RS | p value |  | LPS | RS | p value |  | LPS | RS | p value |  |  |  |
| All cases | 130 | 92 |  |  | 115 | 70 |  |  | 76 | 21 |  |  | 25 | 8 |  |  |  |  |
| Surgical procedures | |  | 0.957 |  |  |  | 0.678 |  |  |  | 0.859 |  |  |  | 0.590 |  | 0.533 | 0.917 |
| TRH | 2 (1.5) | 1 (1.1) |  |  | 1 (0.9) | 0 (0) |  |  | 1 (1.3) | 0 (0) |  |  | 2 (8.0) | 0 (0) |  |  |  |  |
| TRH + BSO/MSO | 124 (95.4) | 88 (95.7) |  |  | 109 (94.8) | 66 (94.3) |  |  | 72 (94.7) | 20 (95.2) |  |  | 22 (88.0) | 8 (100) |  |  |  |  |
| TRH ± BSO/MSO + Omentectomy | 4 (3.1) | 3 (3.3) |  |  | 5 (4.3) | 4 (5.7) |  |  | 3 (3.9) | 1 (4.8) |  |  | 1 (4.0) | 0 (0) |  |  |  |  |
| Lymphadenectomy |  |  | **<0.0001** |  |  |  | **<0.0001** |  |  |  | **<0.0001** |  |  |  | 0.194 |  | 0.083 | 0.434 |
| Not performed | 70 (53.8) | 25 (27.2) |  |  | 73 (63.5) | 21 (30.0) |  |  | 53 (69.7) | 6 (28.6) |  |  | 19 (76.0) | 5 (62.5) |  |  |  |  |
| Sentinel lymph node | 0 (0) | 7 (7.6) |  |  | 0 (0) | 9 (12.9) |  |  | 0 (0) | 4 (19.0) |  |  | 0 (0) | 1 (12.5) |  |  |  |  |
| Pelvic | 56 (43.1) | 55 (59.8) |  |  | 40 (34.8) | 36 (51.4) |  |  | 19 (25.0) | 11 (52.4) |  |  | 6 (24.0) | 2 (25.0) |  |  |  |  |
| Pelvic and aortic | 4 (3.1) | 5 (5.4) |  |  | 2 (1.7) | 4 (5.7) |  |  | 4 (5.3) | 0 (0) |  |  | 0 (0) | 0 (0) |  |  |  |  |
| Estimated blood loss, mL Ɨ |  |  | 0.676 |  |  |  | 0.805 |  |  |  | 0.942 |  |  |  | 0.409 |  | 0.183 * | 0.540 * |
| Mean (standard deviation) | 65.3 (58.7) | 63.9 (66.7) |  |  | 74.6 (54.2) | 111.9 (159.9) |  |  | 83.7 (52.7) | 81.2 (66.8) |  |  | 82.8 (56.5) | 57.5 (46.8) |  |  |  |  |
| Median (min-max) | 50 (0-400) | 50 (0-400) |  |  | 75 (5-250) | 50 (0-800) |  |  | 99 (5-200) | 99 (10-200) |  |  | 99 (9-200) | 50 (10-150) |  |  |  |  |
| Operative time, min ŧ |  |  | **<0.0001** |  |  |  | **<0.0001** |  |  |  | **0.003** |  |  |  | **0.040** |  | 0.259 * | 0.750 * |
| Mean (standard deviation) | 131 (68.2) | 179.4 (75.3) |  |  | 116.6 (55.8) | 178.9 (79.2) |  |  | 120.6 (53.8) | 170 (72.8) |  |  | 104.4 (50.0) | 154.6 (58.6) |  |  |  |  |
| Median (min-max) | 110 (35-389) | 180 (45-480) |  |  | 105 (35-298) | 164 (25-530) |  |  | 100 (50-245) | 150 (75-350) |  |  | 100 (40-240) | 160 (75-235) |  |  |  |  |
| Hospital stay, days ǂ |  |  | 0.066 |  |  |  | **<0.0001** |  |  |  | **0.015** |  |  |  | 0.282 |  | **0.007 *** | 0.439 * |
| Mean (standard deviation) | 3.8 (2.5) | 3.3 (2.1) |  |  | 4.5 (3.4) | 2.9 (1.4) |  |  | 4.4 (1.7) | 4.6 (6.2) |  |  | 4.2 (2.1) | 3.2 (1.2) |  |  |  |  |
| Median (min-max) | 3 (1-21) | 3 (1-12) |  |  | 4 (1-32) | 3 (1-9) |  |  | 4 (1-8) | 3 (2-31) |  |  | 4 (1-9) | 3 (2-5) |  |  |  |  |
| Laparotomic conversion ** | 1 (0.8) | 3 (3.3) | 0.169 |  | 2 (1.7) | 2 (2.9) | 0.612 |  | 2 (2.6) | 1 (4.8) | 0.618 |  | 0 (0) | 0 (0) | - |  | 0.658 | 0.927 |
| Patients with intra-operative complication | 2 (1.5) | 1 (1.1) | 0.774 |  | 1 (0.9) | 3 (4.3) | 0.121 |  | 3 (3.9) | 0 (0) | 0.355 |  | 0 (0) | 0 (0) | - |  | 0.368 | 0.438 continue |
| Patients with post-operative complication within 30 days from surgery | 4 (3.1) | 6 (6.5) | 0.223 |  | 7 (6.1) | 0 (0) | **0.035** |  | 9 (11.8) | 3 (14.3) | 0.763 |  | 2 (8.0) | 0 (0) | 0.409 |  | 0.097 | 0.032 |
| Patients with post-operative complication beyond 30 days from surgery | 2/129 (1.6) | 2/90 (2.2) | 0.715 |  | 5/115 (4.3) | 3/70 (4.3) | 0.984 |  | 4/76 (5.3) | 1/21 (4.8) | 0.927 |  | 0/24 (0) | 0/8 (0) | - |  | 0.326 | 0.806 |
| Adjuvant therapy |  |  | 0.218 |  |  |  | 0.249 |  |  |  | 0.780 |  |  |  | 0.208 |  | 0.363 | **0.024** |
| No | 66 (50.8) | 39 (42.4) |  |  | 59 (51.3) | 42 (60.0) |  |  | 46 (60.5) | 12 (57.1) |  |  | 16 (64.0) | 7 (87.5) |  |  |  |  |
| Yes | 64 (49.2) | 53 (57.6) |  |  | 56 (48.7) | 28 (40.0) |  |  | 30 (39.5) | 9 (42.9) |  |  | 9 (36.0) | 1 (12.5) |  |  |  |  |
| Type of adjuvant therapy † |  |  | 0.877 |  |  |  | 0.161 |  |  |  | 0.092 |  |  |  | 0.349 |  | 0.493 | 0.893 |
| CHT | 15/63 (23.8) | 9/53 (17.0) |  |  | 8/56 (14.3) | 6/28 (21.4) |  |  | 5/30 (16.7) | 0/9 (0) |  |  | 1/9 (11.1) | 1/1 (100) |  |  |  |  |
| EBRT | 18/63 (28.6) | 17/53 (32.1) |  |  | 28/56 (50.0) | 8/28 (28.6) |  |  | 14/30 (46.7) | 2/9 (22.2) |  |  | 4/9 (44.4) | 0/1 (0) |  |  |  |  |
| BRT | 11/63 (17.5) | 10/53 (18.9) |  |  | 11/56 (19.6) | 5/28 (17.9) |  |  | 7/30 (23.3) | 2/9 (22.2) |  |  | 1/9 (11.1) | 0/1 (0) |  |  |  |  |
| CHT+EBRT | 9/63 (14.3) | 9/53 (17.0) |  |  | 3/56 (5.4) | 5/28 (17.9) |  |  | 2/30 (6.7) | 3/9 (33.3) |  |  | 2/9 (22.2) | 0/1 (0) |  |  |  |  |
| CHT+BRT | 0/63 (0) | 1/53 (1.9) |  |  | 0/56 (0) | 1/28 (3.6) |  |  | 0/30 (0) | 0/9 (0) |  |  | 0/9 (0) | 0/1 (0) |  |  |  |  |
| EBRT+BRT | 8/63 (12.7) | 5/53 (9.4) |  |  | 6/56 (10.7) | 3/28 (10.7) |  |  | 2/30 (6.7) | 2/9 (22.2) |  |  | 1/9 (11.1) | 0/1 (0) |  |  |  |  |
| CHT+EBRT+BRT | 2/63 (3.2) | 2/53 (3.8) |  |  | 0/56 (0) | 0/28 (0) |  |  | 0/30 (0) | 0/9 (0) |  |  | 0/9 (0) | 0/1 (0) |  |  |  |  |
| Recurrences | 17 (13.1) | 16 (17.4) |  |  | 19 (16.5) | 5 (7.1) |  |  | 13 (17.1) | 3 (14.3) |  |  | 3 (12.0) | 1 (12.5) |  |  |  |  |
| Deaths | 21 (16.2) | 11 (12) |  |  | 29 (25.2) | 8 (11.4) |  |  | 19 (25) | 3 (14.3) |  |  | 8 (32.0) | 1 (12.5) |  |  |  |  |
| Median FU (95% CI), months § | 40.3 (32.4-49.9) | 30.0 (23.9-39.2) | nc |  | 59.8 (37.1-66.5) | 23.9 (17.6-28.1) | nc |  | 60.1 (34.1-62.7) | 25.6 (8.8-31.6) | nc |  | 75.9 (10.9-80.9) | 12.7 (2.9-35.8) | nc |  | nc | nc |
| Results are presented as n (%) except where indicated. p value was calculated with two sided Pearson's Chi Square test or Mann-Whitney U test for categorical and continuous not normally distributed characteristics respectively, except where indicated. Bold font highlights statistically significant difference. LPS: Laparoscopic Surgery. RS: Robotic Surgery. TRH: Total Radical Hysterectomy. BSO: Bilateral Salpingo-Oophorectomy. MSO: Monolateral Salpingo-Oophorectomy. CHT: Chemotherapy. EBRT: External brachytherapy. BRT: Brachytherapy: AWD: Alive with disease. NED: No evidence of disease. FU: follow up. CI: Confidence interval. nc: not calculated. * Calculated with two sided Kruskall-Wallis test (not normally distributed characteristic). Ɨ Information available for 442/537 patients. ŧ Information available for 517/537 patients. ǂ Information available for 486/537 patients. ** One patient of 82 years old was converted from robotic to laparoscopic surgery for obesity reason. † In one case the type of adjuvant therapy was not available. § Calculated with the inverse Kaplan-Meier technique. | | | | | | | | | | | | | | | | | | |
